# Alexithymia and Apathy in Parkinson’s Disease: Neurocognitive Correlates

**DOI:** 10.3233/BEN-129021

**Published:** 2013

**Authors:** Yelena Bogdanova, Alice Cronin-Golomb

**Affiliations:** ^1^Psychology Research, VA Boston Healthcare System, Boston, MAUSA; ^2^Department of Psychology, Boston University, Boston, MAUSA; ^3^Department of Psychiatry, Boston University School of Medicine, Boston, MAUSA; ^4^Department of Psychiatry, Harvard Medical School, Boston, MAUSA

**Keywords:** Non-motor symptoms, basal ganglia, ACC, frontostriatal, right hemisphere

## Abstract

Non-motor symptoms such as neuropsychiatric and cognitive dysfunction have been found to be common in Parkinson’s disease (PD) but the relation between such symptoms is poorly understood. We focused on alexithymia, an impairment of affective and cognitive emotional processing, as there is evidence for its interaction with cognition in other disorders. Twenty-two non-demented PD patients and 22 matched normal control adults (NC) were administered rating scales assessing neuropsychiatric status, including alexithymia, apathy, and depression, and a series of neuropsychological tests. As expected, PD patients showed more alexithymia than NC, and there was a significant association between alexithymia and disease stage. Alexithymia was associated with performance on non-verbally mediated measures of executive and visuospatial function, but not on verbally mediated tasks. By contrast, there was no correlation between cognition and ratings of either depression or apathy. Our findings demonstrate a distinct association of alexithymia with non-verbal cognition in PD, implicating right hemisphere processes, and differentiate between alexithymia and other neuropsychiatric symptoms in regard to PD cognition.

## 1. Introduction

Parkinson's disease (PD) is traditionally characterized as a motor disorder, but the existence of non-motor symptoms is attracting increasing attention from clinicians and researchers because of their impact on patients' quality of life. Among others, the non-motor symptoms include cognitive dysfunction [1–4] and neuropsychiatric symptoms such as apathy [5–13], depression [14–19], and alexithymia [20,21]. PD-associated cognitive impairments have been attributed to reduced dopaminergic innervation in the nigrostriatal and mesocortical dopaminergic systems, leading to dysfunction of neural circuits including the basal ganglia and cortical projection areas (reviewed in [3]). PET studies have shown decreased network activity [22] and metabolic abnormalities [23] in prefrontal and parietal brain regions in PD patients with mild cognitive impairment, consistent with the cognitive domains commonly affected in PD: executive and visuospatial function [24].

Alexithymia, an impairment of affective and cognitive emotional processing, is a common neuropsychiatric concomitant of PD [25], with an estimated prevalence of 21% in non-demented patients [21,26]. Dopamine depletion in brain areas critical for emotional cognition, such as anterior cingulate cortex (ACC) and orbitofrontal cortex [27,28], may underlie the manifestation of alexithymia in this disorder. Though understudied, it has been suggested that alexithymia should be considered and treated as an important non-motor

ISSN 0953-4180/13/$27.50 © 2013 – IOS Press and the authors. All rights reserved symptom of PD [20]. The multifaceted construct of alexithymia is characterized by the following: a) difficulty identifying and distinguishing between feelings and bodily sensations of emotional arousal, b) difficulty describing feelings, c) reduced imaginal processes, and d) a stimulus-bound, externally-oriented cognitive style [29]. Alexithymia characteristics reflect deficits in the cognitive processing and regulation of emotions, such as the inability to apply adaptive processes for affect regulation (e.g., modulating arousal, expressing or suppressing emotions, and cognitive assimilation), which may in turn contribute to the development and course of various psychiatric and medical disorders, such as depression and diabetes [30–32], and affect treatment outcome [33–35].

According to a current model, alexithymia derives from dysfunction of frontal areas [36,37], specifically the ACC and prefrontal cortex [38]. Support for this model comes from neuroimaging studies [39–43], which also have related alexithymia to predominantly right hemisphere dysfunction. Scores on the standard measure of alexithymia, the Toronto Alexithymia Scale (TAS-20 total), were reported to be significantly correlated with gray matter volume of the right, but not left, pregenual ACC in a sample of healthy older adults [44]. In another study of normal aging, a significant correlation was found between right rostral ACC volume and alexithymia ratings, with more advanced age corresponding to higher alexithymia ratings and to smaller overall right ACC total gray matter volume in rostral and dorsal subregions as well as in the left rostral subregion [45]. Alexithymia has also been related to right-hemisphere lesions in individuals with stroke [46].

There is little known in regard to the relation between alexithymia and cognitive dysfunction in PD. In one study alexithymia was associated with poor performance on a cognitive (visuospatial) test [47]. In individuals with HIV, which is like PD in that it affects frontostriatal areas, associations were documented between alexithymia, but not depression, and performance on measures of executive and visuospatial function [48].

While alexithymia is often associated with depression [49–51], previous research has indicated that they should be considered distinct constructs [52–55]. In PD, some studies also related alexithymia and depression [26,56], while others showed that the two conditions may be reliably dissociated in this disorder [20, 21], suggesting that alexithymia is at least partly independent of depression in PD and may be directly related to the disease process [20].

Apathy, like alexithymia, is associated with damage to the frontal lobes and disruption of the frontal-subcortical anterior cingulate circuits [57–59]. Neuroimaging research has shown that the severity of apathy is associated with the volume of the nucleus accumbens, one of the central structures in the anterior cingulate frontal-subcortical circuit [60]. Apathy, like alexithymia, also appears to be more common in patients with right- than left-hemisphere damage [9,61]. Neuroimaging research has shown that apathy is associated with decreased gray matter volume in the right anterior cingulate in older adults [62]. In PD, apathy was correlated with low gray matter density in bilateral inferior frontal gyrus and inferior parietal gyrus, right cingulate and right precuneus in one study [63]; and with low volume of the right medial temporal lobe in another study [5]. Also in PD, apathy has been related to emotional cognition, specifically the ability to attribute emotional states to others [64]. Similarly, apathy has been related to alexithymia in patients with frontostriatal dysfunction arising from neurodegenerative disease [44] and HIV [48]. The anatomical overlap in neural networks involved in alexithymia and apathy, together with the neuropsychological findings, raises the question of potential overlap in the expressions of alexithymia and apathy in PD.

The present study aimed to explore the expression of neuropsychiatric symptoms in PD without dementia by assessing the effect of neuropsychiatric status, and alexithymia in particular, on cognitive function. Examining the relation between emotional processing and cognitive function is important from both clinical and theoretical perspectives, as it may provide insights into the interaction of emotional and cognitive neural systems. The present study had two main aims. The first was to explore the association between alexithymia and cognition in PD and to consider the mechanisms underlying an association. The ACC connections with the prefrontal and parietal cortices are important for both cognitive (executive in particular) and emotional function [65–67]. We hypothesized that alexithymia level should be associated with cognitive dysfunction associated with these brain areas, including attention, executive function, and visuospatial processing. To address an ongoing debate in the literature regarding lateralized (left vs. right hemisphere) expression of alexithymia and follow the lead of one neuropsychological study of PD that suggested an association between alexithymia and cognitive abilities mediated by the right hemisphere (i.e., visuospatial function) [47], in the present study we related alexithymia to material-specific cognitive domains (verbal vs. non-verbal). We also explored the relation between cognitive performance and various aspects (factors) of alexithymia in PD, as neuroimaging studies have suggested differentiation between neural systems associated with specific aspects of alexithymia. The second aim was to examine the potential overlap between alexithymia and apathy in PD. Based on the results of imaging and behavioral studies as described above, we hypothesized that if apathy and alexithymia share common neurophysiological substrates, there should be an association between the levels of apathy and alexithymia. We also examined the extent of overlap between alexithymia and apathy in regard to their relation to cognition in PD.

## 2. Methods

### 2.1. Participants

Twenty-two consecutively-enrolled non-demented individuals with idiopathic PD (10 men, 12 women) and 22 normal control adults (NC) (10 men, 12 women) matched on socio-demographic variables participated in the study. All participants scored 28 or higher on the Mini-Mental State Examination (MMSE) [68] and were not demented. PD participants were recruited from the Parkinson Clinic of the Department of Neurology, Boston Medical Center and through local support groups. NC were recruited from the community. The research was approved by Boston University's Institutional Review Board. Participants were required to be native speakers of English. Exclusion criteria included co-existing cancer, serious cardiac disease, other serious chronic medical illness, prior intracranial surgery, history of traumatic brain injury, psychiatric diagnosis (exclusive of depression or anxiety) or neurological diagnoses other than PD; history of alcoholism or other drug abuse; history of eye disease or other abnormalities; and use of psychoactive medications, except for use of antidepressants and anxiolytics in the PD group, which are commonly prescribed. PD clinical staging was determined by the patient's neurologist based on the widely used index of motor disability, the Hoehn and Yahr scale (H&Y) [69]. All PD participants were stages I–III (mild to moderate bilateral). The average duration of disease was 7.8 years (SD = 3.1). PD diagnosis was made by patients' neurologists, using UK Parkinson's Disease Society Brain Bank clinical diagnostic criteria [70]. Side of motor symptom onset was obtained by patient report and from neurologist records. Eleven patients had initial motor symptom on the right body side (RPD: 5 men, 6 women) and 11 had left-side onset (LPD: 5 men, 6 women). RPD and LPD groups did not differ in age, education, MMSE, H&Y stage, or disease duration. There were no group differences between male and female PD participants for age, education, MMSE, H&Y stage, or disease duration. All PD participants received daily dopamine replacement therapy and /or dopamine receptor agonists. Levodopa equivalent dosages (LED) were calculated based on previous reports with LED: (regular levodopa dose $\times$ 1) + (levodopa controlled-release dose $\times$ 0.75) + (pramipexole dose $\times$ 67.0) + (ropinirole dose $\times$ 16.67) + (rotigotine $\times$ 16.67) + (pergolide dose and cabergoline dose $\times$ 67.0) + (bromocriptine dose $\times$ 10) + ([regular levodopa dose + levodopa controlled-release dose $\times$ 0.75] $\times$ 0.25, if taking tolcapone or entacapone) [71]. None of the PD participants were taking anticholinergic medications. Three were taking some form of sleep medication. PD participants were tested while on their anti-parkinsonian medications (in their "on" state).

### 2.2. Procedure

Study participants were administered standardized measures of neuropsychiatric functioning and a series of standardized neuropsychological measures sensitive to PD-associated cognitive impairments. The cognitive tests were chosen to sample both verbal and non-verbal domains. Because the PD and NC groups were matched on age, education, and male:female ratio, we compared and report raw scores for all tests. Before participating, each individual provided written informed consent.

#### 2.2.1. Neuropsychiatric status assessment

The 20-item *Toronto Alexithymia Scale* (*TAS-20*) was administered to evaluate alexithymia [72]. It comprises three subscales that reflect distinct facets of alexithymia: Factor 1 (difficulty identifying feelings and distinguishing them from bodily sensations of emotion, DIF), Factor 2 (difficulty describing feelings, DDF), and Factor 3 (externally oriented thinking, EOT). Item responses were rated on a five-point Likert scale ranging from 1 to 5. Higher scores reflect more severe alexithymia. Based on the total score, individuals are categorized as non-alexithymic (scores ranging from 20 to 51), borderline alexithymic (scores ranging from 52 to 60), or alexithymic (scores $\geqslant$ 61) [72].

Apathy was assessed using the modified 14-item *Apathy Scale* (*AS*) [10,73]. Items are rated on a 0-to-3 Likert scale. Scores range from 0 to 42, with higher scores indicative of more severe apathy. Total score was the dependent measure. AS and its modified version were reported to have excellent psychometric properties and have been used to study PD [10].

*The Beck Depression Inventory, Second Edition* (*BDI-II*) [74] is a 21-item self-report instrument that assesses the existence and severity of symptoms of depression as listed in the American Psychiatric Association's Diagnostic and Statistical Manual of Mental Disorders, Fourth Edition [DSM-IV; [75]]. There is a four-point scale for each item ranging from 0 to 3. Total score in the range of 0–13 is considered indicative of minimal depression, 14–19 is mild, 20–28 is moderate, and 29–63 is severe. The BDI is considered a reliable and valid measure of depression in PD [76].

*2.2.2. Cognitive functioning assessment*

The neuropsychological series included a number of tests that we expected to be sensitive to frontostriatal and parietal dysfunction (attention, executive function, visuospatial ability) [3,8,48]. A focus of interest was on the potential dissociation between correlations of neuropsychiatric status and performance on the verbally and non-verbally mediated cognitive tests.

*2.2.2.1. Verbal domain*

*Controlled Oral Word Association Test* [77]. This is a test of verbal fluency: (1) phonemic fluency, in which participants were required to generate words beginning with a particular letter (F, A, S); and (2) category fluency, in which participants generate words that belong to a particular category (Animals). Total number correct within a 60-s time period for each condition was recorded.

*Digit Span*, Wechsler Memory Scale III [78], is a measure of the efficiency of attention (Forward Span) and working memory (Backward Span) in the verbal domain [79]. The total score was used for the group comparison.

*Subtracting from 100 by 7* [80] is a task of sequential arithmetic operation. A neuroimaging study demonstrated bilateral prefrontal and posterior parietal cortex activation during silent subtraction by sevens in healthy adults [81]. The task is presented verbally and requires a spoken verbal answer. Time to completion and number of errors was recorded.

*2.2.2.2. Non-verbal domain*

*The Trail Making Test* (*TMT*) [82] is a test of psychomotor speed, attention, and executive functioning that consists of two subtests. *Trails A* is a test of simple attention and psychomotor speed, in which participants connect numbered circles in ascending order (1-2-3, etc.). *Trails B* is a measure of combined visual search, psychomotor speed, and cognitive flexibility, assessing the ability to shift and maintain the response set. Participants sequentially alternate between alphanumeric sequences (1-A-2-B, etc.). Time to completion and number of errors were recorded.

*Raven's Coloured Progressive Matrices* (*RCPM*) [83]. This is a measure of visuospatial skills and reasoning ability. The task is to choose one of six possible completions of an incomplete pattern matrix. Total score (the number correct out of 36 items) was recorded.

*Clock reading test* [80]. Participants are asked to identify and mark the time shown on each of the 10 clocks presented on a standard paper. Time to completion and number of errors were recorded.

*Visual symbol search test* [84] provides a measure of visual scanning abilities and sustained attention. Participants search and cancel the target symbol in the non-verbal array. Time to completion was recorded.

*Boston Visuospatial Quantitative Battery* (BVSQB; [85]). On the *Drawing to Command* subtest, the participant is required to draw six objects, one at a time. Total number of correct points was recorded. *Right-Left Orientation* subtest requires the identification of right and left on 20 different body parts and objects drawn in various positions. The number of errors was recorded.

*2.2.3. Statistical analyses*

To analyze differences between the PD and NC groups, independent samples $t$-tests (2-tailed) were used. Alpha was set to 0.01 to account for multiple comparisons. Analyses of performance of men and women in the PD and NC groups revealed no significant differences in neuropsychological profile or mood ratings, and data were accordingly collapsed across these subgroups. Pearson correlations were performed to examine associations between neuropsychiatric status and neuropsychological performance. Linear regression analyses were then performed to examine the role of neuropsychiatric and neuropsychological factors in predicting alexithymia in PD, with alexithymia as the dependent variable and neuropsychiatric test scores (apathy and depression) and neuropsychological test scores (those correlated with alexithymia) as independent variables.

## 3. Results

This study used a mixed design, with each participant receiving all assessments. The results are divided

Table 1
Demographic and clinical characteristics of PD and NC participants. Means (SD) are reported unless otherwise indicated

| | NC | PD | *p* |
|---|---|---|---|
| N | 22 | 22 | |
| Age (years) | 61.0 (7.8) | 61.8 (6.1) | ns |
| Education (years) | 16.6 (1.8) | 15.6 (2.3) | ns |
| Disease duration (years) | n/a | 7.8 (3.1) | |
| Hoehn and Yahr stage^ | n/a | 2 (1–3) | |
| LED | n/a | 626.3 (258.5) | |
| Dopamine agonist (%) | n/a | 68.1% | |
| Neuropsychiatric status: | | | |
| TAS-20 total | 32.1 (5.8) | 42.2 (10.3) | *0.0001* |
| TAS-20 Factor 1 | 9.9 (3.2) | 12.3 (4.7) | ns |
| TAS-20 Factor 2 | 8.2 (2.5) | 11.6 (3.5) | *0.001* |
| TAS-20 Factor 3 | 14.4 (4.2) | 18.2 (5.1) | *0.009* |
| AS total | 7.8 (4.0) | 8.1 (4.1) | ns |
| BDI-II total | 4.2 (3.9) | 6.9 (3.6) | ns |

^Median (range); n/a: not applicable; LED: Levodopa equivalent dosage; TAS-20: Toronto Alexithymia Scale-20; AS: Apathy Scale; BDI-II: Beck Depression Inventory-II.

into two sections. The first section presents findings regarding the effects of PD on neuropsychiatric status and cognitive function, and the second section relates neuropsychiatric status to cognitive performance, specifically to the verbal vs. non-verbal cognitive domains.

### 3.1. *Effects of PD on neuropsychiatric status and cognitive function*

#### 3.1.1. *Neuropsychiatric status*

Independent groups $t$-tests revealed that the PD participants reported significantly higher alexithymia levels (TAS-20 total) than the NC group. There were no group differences in depression or apathy level, though there was a trend toward more depression in the PD group (Table 1). Comparison of the LPD and RPD subgroups revealed no significant differences in alexithymia, apathy or depression levels [all $p's > 0.61$].

We also explored the relation between alexithymia factors and both apathy and depression using Pearson correlations (two-tailed). Alexithymia ratings (TAS-20) correlated with apathy and depression. Specifically, F1 was associated with depression [$r = 0.36$, $p < 0.018$], whereas F3 was associated with apathy [$r = 0.36$, $p < 0.017$] in PD. We observed a trend toward a similar pattern of correlations (F1 with depression and F3 with apathy level) [$p's < 0.052$] in NC.

#### 3.1.2. *Cognitive performance*

To examine whether the PD group exhibited cognitive deficits compared to the NC group, we conducted independent groups $t$-tests. The PD group performed

Table 2
Neuropsychological performance. Raw score mean values (SD)

| | NC | PD | *p* |
|---|---|---|---|
| *Verbal domain* | | | |
| FAS total | 53.5 (15.5) | 42.1 (8.8) | *0.005* |
| Animals total | 21.8 (3.4) | 19.4 (4.4) | ns |
| Digit span forward total | 12.2 (2.2) | 11.0 (1.9) | ns |
| Digit span backward total | 8.9 (2.6) | 6.7 (2.2) | *0.004* |
| 100-7 time (sec) | 43.8 (22.4) | 69.5 (38.0) | *0.01* |
| 100-7 errors | 1.1 (1.6) | 2.0 (1.8) | ns |
| *Non-Verbal domain* | | | |
| TMT A errors | 0.0 (0.0) | 0.1 (0.2) | ns |
| TMT B errors | 0.3 (0.6) | 0.5 (1.2) | ns |
| RCPM total | 34.1 (2.3) | 31.6 (3.7) | *0.01* |
| Clock reading time (sec) | 47.4 (18.4) | 62.2 (19.9) | *0.01* |
| Clock reading errors | 0.9 (1.5) | 2.1 (7.1) | ns |
| Visual symbol search time (sec) | 83.2 (23.4) | 133.6 (75.8) | *0.005* |
| BVSQB drawing total correct | 11.0 (2.0) | 9.4 (2.1) | *0.01* |
| BVSQB right-left errors | 3.9 (2.2) | 5.2 (3.1) | ns |

FAS: Controlled Oral Word Association Test (F, A, S); TMT: Trail Making Test; RCPM: Raven's Coloured Progressive Matrices; BVSQB: Boston Visuo-spatial Quantitative Battery.

more poorly than the NC group on a number of tests, as expected. Significant group differences were observed on verbal and non-verbal measures of executive and visuospatial functioning (see Table 2).

### 3.2. *Relation between neuropsychiatric status and cognitive performance*

To explore the nature of the relation between alexithymia and lateralized cognitive functions in PD, we performed Pearson correlations between alexithymia factors and neuropsychological performance in the verbal and non-verbal domains. Because we expected more severe alexithymia to be associated with poorer executive function and visuospatial abilities, one-tailed tests were used. To achieve a reasonable balance between Type I and Type II errors and to allow the examination of potential relations among variables, we adopted a more conservative significance level of 0.025. This adjustment was made for similar reasons in our previous study of alexithymia in another frontostriatal disorder, HIV [48]. As predicted, alexithymia was associated with performance on a number of *non-verbally* mediated executive function and visuospatial measures [TMT A, TMT B, Clock Reading Test, Visual Symbol Search, and BVSQB Drawing Test] (Table 3). By contrast there were no significant correlations between alexithymia and performance on *verbally* mediated neuropsychological measures (all $r's < 0.4$; all $p's > 0.1$). We found no significant correlations between alexithymia and neuropsychological performance in the NC group

Table 3
Correlations between alexithymia factor scores and performance on non-verbal neuropsychological tests in PD

| | TAS-20 Factor 1 | TAS-20 Factor 2 | TAS-20 Factor 3 |
|---|---|---|---|
| TMT A errors | *0.667**** | ns | ns |
| TMT B errors | ns | ns | *0.529*** |
| RCPM total | ns | ns | ns |
| Clock reading errors | ns | ns | *0.435** |
| Visual symbol search time | ns | *0.434** | ns |
| BVSQB drawing total correct | *−0.535*** | ns | ns |
| BVSQB right-left errors | *0.523*** | ns | ns |

TAS-20: Toronto Alexithymia Scale-20; Factor 1: difficulty identifying feelings; Factor 2: difficulty describing feelings; Factor 3: externally oriented thinking; FAS: Controlled Oral Word Association Test (F, A, S); TMT: Trail Making Test; RCPM: Raven's Coloured Progressive Matrices; BVSQB: Boston Visuo-spatial Quantitative Battery; Note: There were no significant correlations between alexithymia factor scores and performance on neuropsychological tests in the verbal domain. *$p < 0.25$, **$p < 0.01$, **$p < 0.001$.

(all $r's < 0.3$; all $p's > 0.15$). There were no correlations between apathy (AS) or depression (BDI-II) ratings and neuropsychological performance for either group (all $r's < 0.3$; all $p's > 0.17$).

To further examine the role of neuropsychiatric and neuropsychological factors in predicting alexithymia (TAS-20 total) in PD, we performed multiple regression analyses. The first variable entered into the equation was apathy (AS) [slope = 0.542, $R^2 = 0.294$, $t(20) = 2.4$, $p = 0.03$], indicating an expected increase of 0.54 in TAS-20 total score for each point increase on AS. In the second step, TMT A (errors) was entered and significantly contributed to predicting the level of alexithymia [slope = 0.763, $R^2$ change = 0.289, $t(19) = 3.0$, $p = 0.01$]. Patients who had more errors on TMT A showed a higher alexithymia level, with expected increase of 0.76 for each point increase on TMT A errors.

We also assessed the association between degree of alexithymia and disease stage (H&Y) using multiple Spearman's correlations (two-tailed). Alexithymia ratings significantly correlated with disease stage as shown for the overall alexithymia rating (TAS-20 total: $r = 0.56$, $p < 0.020$) and for F3 specifically (externally oriented thinking) ($r = 0.59$, $p < 0.013$). By contrast, there were no significant correlations between H&Y stage and severity of apathy (AS) or depression (BDI-II) (all $r's < 0.39$; all $p's > 0.12$).

## 4. Discussion

We examined the effect of PD on alexithymia and its relation to cognition in non-demented individuals with PD. First, as expected, we found the expected PD-related deficits in verbal and non-verbal cognitive domains, including attention, executive function and visuospatial function, which is reflective of frontostriatal and parietal dysfunction associated with the disorder. Second, also as predicted, PD patients reported significantly higher levels of alexithymia than the NC group. The group differences applied to the total alexithymia score and to two of the three alexithymia processing components, F2 (difficulty describing feelings) and F3 (externally oriented thinking). The extent of alexithymia and each of its processing components (F1, F2, and F3) but not the extent of depression or apathy, significantly correlated with performance on neuropsychological measures of attention, executive and visuospatial function in this sample. There was a differential association between alexithymia and performance of tests in the non-verbal, but not the verbal, cognitive domain, supporting our hypothesis that alexithymia is associated with cognitive deficits mediated predominantly by the right hemisphere. The results are in accord with those of a study that showed an association of alexithymia and performance on a visuospatial task in a hospitalized non-demented PD patient sample [47]. To our knowledge, ours is the first study to demonstrate a differential association of alexithymia and apathy with cognitive dysfunction in PD in relation to the lateralized cognitive domains. While there was a significant association between alexithymia factors, apathy and depression in PD, neither apathy nor depression level correlated with cognitive performance.

### 4.1. Potential brain substrates of alexithymia in PD

The extent of alexithymia significantly correlated with attention, executive and visuospatial function, cognitive domains associated with frontostriatal and parietal pathology. This finding supports the current "frontal" alexithymia model, which postulates that alexithymia is the result of dysfunctional mechanisms in the frontal cortex [36–38]. The visuospatial cognitive domain reflecting parietal dysfunction is also affected by PD and was associated with alexithymia in our study. The ACC connections with the prefrontal and parietal cortex are important for both cognitive and emotional function [65–67], which may explain the significant relation between visuospatial dysfunction and the extent of alexithymia in PD.

### 4.2. Apathy and alexithymia in PD

Previous imaging and behavioral research (reviewed in the Introduction) have suggested that apathy and alexithymia may share some common neurophysiological substrates within frontostriatal circuits, and related apathy and alexithymia to right-hemisphere dysfunction. We found that apathy and alexithymia differentially correlated with performance on neuropsychological measures. Whereas alexithymia was significantly associated with performance on non-verbal measures of executive function and visuospatial ability, apathy was not associated with performance on any of the tasks. The fact that apathy and alexithymia can be dissociated in PD implies that they have at least partially distinct neural substrates within frontostriatal circuits.

The observed dissociation between the two neuropsychiatric conditions in relation to cognition suggests at least two potential interpretations: (1) the underlying neural substrates for apathy and alexithymia are differentially affected by PD neuropathological processes; (2) it was too early in the course of the disease to detect the apathy-associated changes in our sample. In line with this argument, we found no differences in apathy levels between our non-demented PD and NC groups, which is consistent with the notion that apathy may be a reflection of more advanced (moderate to severe) stage of PD [25]. There was, however, a significant correlation between apathy level and severity of alexithymia (F3) in our sample. Previous neuroimaging research in healthy older adults has found a significant correlation between right rostral ACC volume and alexithymia ratings (TAS-20) for both total score and F3 [45]. Taken together, these findings suggest a potential overlap in the neural substrates (the rostral subregion of ACC) of apathy and alexithymia, specific to F3.

### 4.3. Depression and alexithymia in PD

We found no significant group difference between PD and NC for depression, though there was a trend for the PD group to be more depressed. The severity of depression correlated with alexithymia level (TAS-20, F1) but did not correlate with performance on any of the cognitive tests, meaning that the neuropsychological abnormalities observed in our sample could not be attributed to depression.

The results of multiple regression analyses further clarified the association between alexithymia, apathy and depression in PD. We found that apathy, but not depression, predicted higher levels of alexithymia. These findings are consistent with previous studies that indicated that although alexithymia may be associated with depression, these neuropsychiatric conditions can be dissociated in patients with PD [20,21].

Of note, in contrast to what was found in our PD group, in our healthy control group there were no correlations between alexithymia (or apathy or depression) and performance on the neuropsychological measures in NC group in our study. It is possible that the interaction between the processing components of alexithymia and cognitive dysfunction occurs only in the presence of cognitive deficits. These results are consistent with our findings of a significant correlation between alexithymia and cognition in patients with HIV but not in their matched healthy control group [48].

### 4.4. PD and the individual processing components of alexithymia

We explored the association between cognition and the individual processing components of alexithymia in PD, as neuroimaging and behavioral studies have suggested distinct neural mechanisms underlying the components. In one study of normal aging, there was a significant correlation between right rostral ACC volume and alexithymia ratings on TAS-20 F3 (Externally Oriented Thinking, EOT) [45]. These findings suggested that deterioration in the rostral subregion of the ACC with age may contribute to alexithymia, and more specifically to F3. In a study of patients with stroke, F1, Difficulty Identifying Feelings (DIF), and F2, Difficulty Describing Feelings (DDF), were related to right-hemisphere lesions [46]. Though limited in number, these studies provided evidence that individual processing components of alexithymia may be associated with distinct brain regions. Specifically, F3 is thought to depend on the rostral ACC. Our results support this notion, as we observed group differences between PD and NC on F3 but not F1. See Bogdanova et al. [48] for further consideration of the role of the ACC in emotional cognition.

Dissociations between cognitive and affective aspects of alexithymia (reflected by alexithymia factors) have been reported previously in clinical and nonclinical populations [86]. F1 (DIF) reflects the affective dimension of alexithymia, and was found to be strongly associated with depression ratings in a clinically depressed group [87], which is consistent with our findings. F3 (EOT, considered the cognitive component) was found to be significantly associated with smaller rostral ACC volume in healthy older adults [45]; with diagnosis of obsessive-compulsive disorder, a fronto-striato-thalamocortical disorder associated with dysfunction of the orbitofrontal cortex, ACC, thalamus and caudate nucleus [87]; and with cognitive performance in another frontostriatal disorder, HIV [48]. In the present study of PD, F3 was likewise associated with cognitive performance, and with apathy level. F2, by contrast, was associated with only one (visuospatial) measure in our study, similar to the observation reported in the HIV study, in which F2 was related to only a subset of non-verbally mediated tasks [48], and consistent with an earlier report of F2 being related to right-hemisphere lesions [46]. We also observed dissociations between alexithymia factors and other neuropsychiatric symptoms. Specifically, apathy was significantly associated with F3 alone, whereas depression was associated with F1 alone, and F2 did not correlate with depression or apathy. Taken together, these findings provide further evidence for the possibility of anatomically and functionally distinct neural substrates for the individual processing components of alexithymia.

### 4.5. Neuropsychiatric status and disease stage

Our results indicated a specific association between neuropsychiatric status and disease stage in non-demented PD. The severity of alexithymia (TAS total and F3 ratings), but not apathy or depression, were significantly correlated with the Hoehn and Yahr index of disease stage. It should be noted that our sample was restricted to individuals with mild to moderate PD, in stages I to III of the disease.

### 4.6. Study limitations

Our sample of PD patients, although large enough to reveal significant differences on the various measures in relation to the control group, was too small to allow us to investigate potential subgroup effects, such as by side of PD motor onset, type of motor symptom at onset (tremor vs. other), or interactions with gender. Our PD group included approximately equal numbers of those with LPD and RPD as well as of men and women, but there was insufficient power to explore potential subgroup effects in any depth. Also, as noted above, we restricted the study to individuals with mild to moderate PD without dementia. Those with more severe disease, with or without dementia, may show a different pattern of results in regard to neuropsychiatric status in general and its relation to cognition in particular.

### 4.7. Implications

The identification and treatment of alexithymia in patients with PD is very important, as alexithymia-related deficits in cognitive processing and the regulation of emotions have been shown to negatively affect treatment outcome in other medical and psychiatric disorders. Apathy is another key symptom to address in PD, as it can have significant impact on quality of life and patient disability [88,89]. Apathy level but not depression was associated with alexithymia severity in our study. Alexithymia and apathy are treatable conditions, and timely screening and intervention may protect the quality of life and reduce disability in individuals with PD. Our results indicated a correlation of alexithymia and disease stage, even in this relatively mildly impaired PD sample (non-demented, Hoehn and Yahr stages I to III only). Longitudinal studies are needed to investigate the temporal dynamics and change in the expression of these neuropsychiatric deficits associated with PD progression, and evaluate the effect of treatment of these neuropsychiatric conditions on cognitive function and quality of life in PD.

## Acknowledgements

This work was supported in part by the Rehabilitation Research and Development Service of the Department of Veterans Affairs, and by grants to Y. Bogdanova (NIH F31 NS052131) and A. Cronin-Golomb (R01NS067128 and R21 NS043730) from the National Institute of Neurological Disorders and Stroke. We thank Dr. Sandy Neargarder for expert advice on statistical analyses; Mirella Diaz-Santos, M.A., and Maria Valmas, M.A., for assistance with data collection; and Sarah M. Kark, B.S., for assistance with manuscript preparation. The authors have no conflicts of interest to disclose.

## References

[1] Zgaljardic DJ, Borod JC, Foldi NS, Mattis P. A review of the cognitive and behavioral sequelae of Parkinson's disease: relationship to frontostriatal circuitry. Cogn Behav Neurol. 2003; 16(4): 193-210.
[2] Amick MM, Schendan HE, Ganis G, Cronin-Golomb A. Frontostriatal circuits are necessary for visuomotor transformation: Mental rotation in Parkinson's disease. Neuropsychologia. 2006; 44(3): 339-349.
[3] Cronin-Golomb A. Parkinson's disease as a disconnection syndrome. Neuropsychol Rev. 2010; 20(2): 191-208.
[4] Davidsdottir S, Wagenaar R, Young D, Cronin-Golomb A. Impact of optic flow perception and egocentric coordinates on veering in Parkinson's disease. Brain. 2008; 131(11): 2882-2893.

[5] Isella V, Melzi P, Grimaldi M, Iurlaro S, Piolti R, Ferrarese C, et al. Clinical, neuropsychological, and morphometric correlates of apathy in Parkinson's disease. Mov Disord. 2002; 17(2): 366-371.
[6] Pluck GC, Brown RG. Apathy in Parkinson's disease. J Neurol Neurosurg Psychiatry. 2002; 73(6): 636-642.
[7] Kirsch-Darrow L, Fernandez HH, Marsiske M, Okun MS, Bowers D. Dissociating apathy and depression in Parkinson disease. Neurology. 2006; 67(1): 33-38.
[8] Bogdanova Y, Cronin-Golomb A. Neurocognitive correlates of apathy and anxiety in Parkinson's disease. Parkinsons Dis. 2012; 2012.
[9] Aarsland D, Larsen JP, Lim NG, Janvin C, Karlsen K, Tandberg E, et al. Range of neuropsychiatric disturbances in patients with Parkinson's disease. J Neurol Neurosurg Psychiatry. 1999; 67(4): 492-496.
[10] Starkstein SE, Mayberg HS, Preziosi TJ, Andrezejewski P, Leiguarda R, Robinson RG. Reliability, validity, and clinical correlates of apathy in Parkinson's disease. J Neuropsychiatry Clin Neurosci. 1992; 4(2): 134-139.
[11] Starkstein SE. The riddle of psychiatric disorders in Parkinson disease: from phenomenology to treatment. Am J Geriatr Psychiatry. 2012; 20(2): 99-103.
[12] Aarsland D, Litvan I, Larsen JP. Neuropsychiatric symptoms of patients with progressive supranuclear palsy and Parkinson's disease. J Neuropsychiatry Clin Neurosci. 2001; 13(1): 42-49.
[13] Levy ML, Cummings JL, Fairbanks LA, Masterman D, Miller BL, Craig AH, et al. Apathy is not depression. J Neuropsychiatry Clin Neurosci. 1998; 10(3): 314-319.
[14] Leentjens AFG. Depression in Parkinson's disease: conceptual issues and clinical challenges. J Geriatr Psychiatry Neurol. 2004; 17(3): 120-126.
[15] McDonald WM, Richard IH, DeLong MR. Prevalence, etiology, and treatment of depression in Parkinson's disease. Biol Psychiatry. 2003; 54(3): 363-375.
[16] Reijnders JS, Ehrt U, Weber WE, Aarsland D, Leentjens AFG. A systematic review of prevalence studies of depression in Parkinson's disease. Mov Disord. 2008; 23(2): 183-189.
[17] Cimino CR, Siders CA, Zesiewicz TA. Depressive symptoms in Parkinson disease: degree of association and rate of agreement of clinician-based and self-report measures. J Geriatr Psychiatry Neurol. 2011; 24(4): 199-205.
[18] Kirsch-Darrow L, Marsiske M, Okun MS, Bauer R, Bowers D. Apathy and depression: separate factors in Parkinson's disease. J Int Neuropsychol Soc. 2011; 17(6): 1058-1066.
[19] Santangelo G, Vitale C, Trojano L, Longo K, Cozzolino A, Grossi D, et al. Relationship between depression and cognitive dysfunctions in Parkinson's disease without dementia. J Neurol. 2009; 256(4): 632-638.
[20] Assogna F, Palmer K, Pontieri FE, Pierantozzi M, Stefani A, Gianni W, et al. Alexithymia Is a Non-Motor Symptom of Parkinson Disease. Am J Geriatr Psychiatry. 2012; 20(2): 133-141.
[21] Costa A, Peppe A, Carlesimo GA, Salamone G, Caltagirone C. Prevalence and Characteristics of Alexithymia in Parkinson's Disease. Psychosomatics. 2010; 51(1): 22-28.
[22] Huang C, Tang C, Feigin A, Lesser M, Ma Y, Pourfar M, et al. Changes in network activity with the progression of Parkinson's disease. Brain. 2007; 130(Pt 7): 1834-1846.
[23] Huang C, Mattis P, Perrine K, Brown N, Dhawan V, Eidelberg D. Metabolic abnormalities associated with mild cognitive impairment in Parkinson disease. Neurology. 2008; 70(16 Pt 2): 1470-1477.
[24] Huber SJ, Bornstein RA, editors. Neuropsychological evaluation of Parkinson's disease. New York: Oxford University Press; 1992.
[25] Poletti M, De Rosa A, Bonuccelli U. Affective symptoms and cognitive functions in Parkinson's disease. J Neurol Sci. 2012; 317(1–2): 97-102.
[26] Costa A, Peppe A, Carlesimo GA, Pasqualetti P, Caltagirone C. Alexithymia in Parkinson's disease is related to severity of depressive symptoms. Eur J Neurol. 2006; 13(8): 836-841.
[27] Bermond B, Vorst HC, Moormann PP. Cognitive neuropsychology of alexithymia: implications for personality typology. Cogn Neuropsychiatry. 2006; 11(3): 332-360.
[28] Lane RD. Neural Correlates of Conscious Emotional Experience. In: Lane RD, Nadel L, editors. Cognitive Neuroscience of Emotion. New York: Oxford University Press; 2000. p. 359–360.
[29] Bagby RM, Taylor GJ. Affect dysregulation and alexithymia. In: Taylor GJ, Bagby RM, Parker JDA, editors. Disorders of affect regulation Alexithymia in medical and psychiatric illness. Cambridge (UK): Cambridge University Press; 1997; pp. 26-45.
[30] Lumley MA, Stettner L, Wehmer F. How are alexithymia and physical illness linked? A review and critique of pathways. J Psychosom Res. 1996; 41(6): 505-518.
[31] Lumley MA, Neely LC, Burger AJ. The Assessment of Alexithymia in Medical Settings: Implications for Understanding and Treating Health Problems. J Pers Assess. 2007; 89(3): 230-246.
[32] Taylor GJ, Bagby RM, Parker JD. Disorders of affect regulation: Alexithymia in medical and psychiatric illness. New York: Cambridge University Press; 1997.
[33] Ogrodniczuk JS, Piper WE, Joyce AS. Effect of alexithymia on the process and outcome of psychotherapy: a programmatic review. Psychiatry Res. 2011; 190(1): 43-48.
[34] Parker JD, Keefer KV, Taylor GJ, Bagby RM. Latent structure of the alexithymia construct: a taxometric investigation. Psychol Assess. 2008; 20(4): 385-396.
[35] Porcelli P, Tulipani C, Maiello E, Cilenti G, Todarello O. Alexithymia, coping, and illness behavior correlates of pain experience in cancer patients. Psychooncology. 2007; 16(7): 644-650.
[36] Davidson RJ, Ekman P, Saron CD, Senulis JA, Friesen WV. Approach-withdrawal and cerebral asymmetry: emotional expression and brain physiology. I. J Pers Soc Psychol. 1990; 58(2): 330-341.
[37] Gainotti G. Disorders of emotions and affect in patients with unilateral brain damage. In: Boller F, Grafman J, editors. Handbook of Neuropsychology. Amsterdam: Elsevier; 1989; pp. 345-358.
[38] Lane RD, Ahern GL, Schwartz GE, Kaszniak AW. Is alexithymia the emotional equivalent of blindsight? Biol Psychiatry. 1997; 42(9): 834.
[39] Borsci G, Boccardi M, Rossi R, Rossi G, Perez J, Bonetti M, et al. Alexithymia in healthy women: A brain morphology study. J Affect Disord. 2009; 114(1–3): 208-215.
[40] Gündel H, Lopez-Sala A, Ceballos-Baumann AO, Deus J, Cardoner N, Marte B. Alexithymia correlates with the size of the right anterior cingulate. Psychosom Med. 2004; 56(6): 609-610.
[41] Huber M, Herholz K, Habedank B, Thiel A, Muller-Kuppers M, Ebel H, et al. Different patterns of regional brain activation during emotional stimulation in alexithymics in comparison with normal controls. Psychother Psychosom Med Psychol. 2002; 52(11): 469-478.

[42] Kano M, Fukudo S, Gyoba J, Kamachi M, Tagawa M, Mochizuki H. Specific brain processing of facial expressions in people with alexithymia: an H215O-PET study. Brain. 2003; 126(6): 1474-1484.

[43] Karlsson H, Naatanen P, Stenman H. Cortical activation in alexithymia as a response to emotional stimuli. Br J Psychiatry. 2008; 192(1): 32-38.

[44] Sturm VE, Levenson RW. Alexithymia in neurodegenerative disease. Neurocase. 2011; 17(3): 242-250.

[45] Paradiso S, Vaidya JG, McCormick LM, Jones A, Robinson RG. Aging and Alexithymia: Association With Reduced Right Rostral Cingulate Volume. Am J Geriatr Psychiatry. 2008; 16(9): 760-769.

[46] Spalletta G, Pasini A, Costa A, De Angelis D, Ramundo N, Paolucci S, et al. Alexithymic Features in Stroke: Effects of Laterality and Gender. Psychosom Med. 2001; 63(6): 944-950.

[47] Costa A, Peppe A, Carlesimo GA, Salamone G, Caltagirone C. Neuropsychological correlates of alexithymia in Parkinson's disease. J Int Neuropsychol Soc. 2007; 13(06): 980-992.

[48] Bogdanova Y, Díaz-Santos M, Cronin-Golomb A. Neurocognitive correlates of alexithymia in asymptomatic individuals with HIV. Neuropsychologia. 2010; 48(5): 1295-1304.

[49] Honkalampi K, Hintikka J, Saarinen P, Lehtonen J, Viinamaki H. Is alexithymia a permanent feature in depressed patients? Psychother Psychosom. 2000; 69(6): 303-308.

[50] Saarijärvi S, Salminen JK, Toikka TB. Alexithymia and depression: A 1-year follow-up study in outpatients with major depression. J Psychosom Res. 2001; 51(6): 729-733.

[51] Müller J, Bühner M, Ellgring H. Relationship and Differential Validity of Alexithymia and Depression: A Comparison of the Toronto Alexithymia and Self-Rating Depression Scales. Psychopathology. 2003; 36(2): 71-77.

[52] Parker JD, Bagby RM, Taylor GJ. Alexithymia and depression: Distinct or overlapping constructs? Compr Psychiatry. 1991; 32(5): 387-394.

[53] Wise TN, Jani NN, Kass E, et al. Alexithymia: relationship to severity of medical illness and depression. Psychother Psychosom. 1988; 50(2): 68-71.

[54] Wise TN, Mann LS, Hill B. Alexithymia and depressed mood in the psychiatric patient. Psychother Psychosom. 1990; 54(1): 26-31.

[55] Wood RL, Williams C. Neuropsychological correlates of organic alexithymia. J Int Neuropsychol Soc. 2007; 13(3): 471-479.

[56] Poletti M, Frosini D, Pagni C, Lucetti C, Del Dotto P, Tognoni G, et al. Alexithymia may modulate decision making in patients with de novo Parkinson's disease. Funct Neurol. 2011; 26(3): 127-131.

[57] Cummings JL. Frontal-subcortical circuits and human behavior. Arch Neurol. 1993; 50(8): 873-880.

[58] Tekin S, Cummings JL. Frontal-subcortical neuronal circuits and clinical neuropsychiatry: an update. J Psychosom Res. 2002; 53(2): 647-654.

[59] Levy R, Dubois B. Apathy and the Functional Anatomy of the Prefrontal Cortex – Basal Ganglia Circuits. Cereb Cortex. 2006; 16(7): 916-928.

[60] Paul RH, Brickman AM, Navia B, Hinkin C, Malloy PF, Jefferson AL, et al. Apathy is associated with volume of the nucleus accumbens in patients infected with HIV. J Neuropsychiatry Clin Neurosci. 2005; 17(2): 167-171.

[61] McAllister TW. Apathy. Semin Clin Neuropsychiatry. 2000; 5(4): 275-282.

[62] Lavretsky H, Ballmaier M, Pham D, Toga A, Kumar A. Neuroanatomical characteristics of geriatric apathy and depression: a magnetic resonance imaging study. Am J Geriatr Psychiatry. 2007; 15(5): 386-394.

[63] Reijnders JS, Scholtissen B, Weber WE, Aalten P, Verhey FR, Leentjens AF. Neuroanatomical correlates of apathy in Parkinson's disease: A magnetic resonance imaging study using voxel-based morphometry. Mov Disord. 2010; 25(14): 2318-2325.

[64] Santangelo G, Vitale C, Trojano L, Errico D, Amboni M, Barbarulo AM, et al. Neuropsychological correlates of theory of mind in patients with early Parkinson's disease. Mov Disord. 2012; 27(1): 98-105.

[65] Bush G, Luu P, Posner MI. Cognitive and emotional influences in anterior cingulate cortex. Trends Cogn Sci. 2000; 4(6): 215-222.

[66] Nieuwenhuis S, Ridderinkhof KR, Blom J, Band GP, Kok A. Error-related brain potentials are differentially related to awareness of response errors: evidence from an antisaccade task. Psychophysiology. 2001; 38(5): 752-760.

[67] Posner MI, DiGirolamo GJ. Executive attention: Conflict, target detection and cognitive control. In: Parasuraman R, editor. The attentive brain. Cambridge (MA): MIT Press; 1998; pp. 401-423.

[68] Folstein MF, Folstein SE, McHugh PR. "Mini-mental state". A practical method for grading the cognitive state of patients for the clinician. J Psychiatr Res. 1975; 12(3): 189-198.

[69] Hoehn MM, Yahr MD. Parkinsonism: onset, progression and mortality. Neurology. 1967; 17(5): 427-442.

[70] Hughes AJ, Daniel SE, Kilford L, Lees AJ. Accuracy of clinical diagnosis of idiopathic Parkinson's disease: a clinico-pathological study of 100 cases. J Neurol Neurosurg Psychiatry. 1992; 55(3): 181-184.

[71] Gjerstad MD, Boeve B, Wentzel-Larsen T, Aarsland D, Larsen JP. Occurrence and clinical correlates of REM sleep behaviour disorder in patients with Parkinson's disease over time. J Neurol Neurosurg Psychiatry. 2008; 79(4): 387-391.

[72] Bagby RM, Parker JDA, Taylor GJ. The twenty-item Toronto Alexithymia Scale-I. Item selection and cross-validation of the factor structure. J Psychosom Res. 1994; 38(1): 23-32.

[73] Marin RS, Biedrzycki RC, Firinciogullari S. Reliability and validity of the Apathy Evaluation Scale. Psychiatry Res. 1991; 38(2): 143-162.

[74] Beck AT, Steer RA, Brown GK. Beck Depression Inventory-II. San Antonio (TX): The Psychological Corporation; 1996.

[75] American Psychiatric Association. Diagnostic and Statistical Manual of Mental Disorders. 4th ed., Washington (DC): American Psychiatric Association; 1994.

[76] Levin BE, Llabre MM, Weiner WJ. Parkinson's disease and depression: psychometric properties of the Beck Depression Inventory. J Neurol Neurosurg Psychiatry. 1988; 51(11): 1401-1404.

[77] Benton AL, Hamsher K. Multilingual Aphasia Examination. 2nd ed., Iowa City (IA): AJA Associates, Inc; 1989.

[78] Wechsler D. The Wechsler Memory Scale-III Manual. 3rd ed., San Antonio (TX): The Psychological Corporation; 1997.

[79] Lezak MD, Howieson DB, Loring DW. Neuropsychological assessment. 4th ed., New York: Oxford University Press; 2004.

[80] Luria AR. Higher Cortical Functions in Man. Moscow: Moscow University Press; 1962.

[81] Rueckert L, Lange N, Partiot A, Appollonio I, Litvan I, Le Bihan D, et al. Visualizing cortical activation during mental calculation with functional MRI. Neuroimage. 1996; 3(2): 97-103.
[82] Armitage SG. An analysis of certain psychological tests used for the evaluation of brain injury. Psychol Monogr. 1946; 60(1, Serial No. 277).
[83] Raven JC. Guide to using the Coloured Progressive Matrices. London: H.K. Lewis; 1965.
[84] Mesulam MM. Attention, confusional state, and attention. In: Mesulam MM, editor. Principles of behavioral neurology. Philadelphia: F.A. Davis Company; 1985.
[85] Goodglass H, Kaplan E. The assessment of aphasia and related disorders. 2nd ed., Philadelphia: Lea and Febiger; 1983.
[86] Larsen JK, Brand N, Bermond B, Hijman R. Cognitive and emotional characteristics of alexithymia: a review of neurobiological studies. J Psychosom Res. 2003; 54(6): 533-541.
[87] Bankier B, Aigner M, Bach M. Alexithymia in DSM-IV disorder: Comparative evaluation of somatoform disorder, panic disorder, obsessive-compulsive disorder, and depression. Psychosomatics. 2001; 42: 235-240.
[88] Antonini A, Barone P, Marconi R, Morgante L, Zappulla S, Pontieri F, et al. The progression of non-motor symptoms in Parkinson's disease and their contribution to motor disability and quality of life. J Neurol. 2012: 1-11.
[89] Lauterbach EC. The neuropsychiatry of Parkinson's disease. Minerva Med. 2005; 96(3): 155-173.
